# The Peculiar Necks of Herons and Anhingas: A Study of Cervical Morphology in Pelecanimorph Birds

**DOI:** 10.1093/iob/obag004

**Published:** 2026-02-12

**Authors:** R C Fleming, C R Black

**Affiliations:** Department of Ecology, Evolution, and Organismal Biology, Brown University, Providence, RI, USA; Smithsonian National Museum of Natural History, Washington, DC, USA

## Abstract

The remarkable diversity in avian neck morphology likely influences neck movement in behaviors such as preening and prey capture. Ardeids (herons, egrets, and bitterns) and anhingas are thought to have evolved specialized mid-neck vertebrae to facilitate their ambush predation motion, which involves a rapid straightening of the neck to propel the head toward prey. Although prior studies have suggested that both groups possess distinctive cervical vertebral morphology, a broader comparative framework incorporating extensive taxonomic sampling, information-rich shape data, and phylogenetic context is needed to evaluate the uniqueness of their neck organization relative to close relatives. Here, we examined cervical vertebral morphology in 24 species across the Pelecanimorphae, with a focus on Ardeidae and Anhingidae. Using three-dimensional geometric morphometrics and phylogenetic comparative methods, we quantified vertebral shape variation and cervical regionalization across the five morphologically distinct regions of the avian neck. We assessed patterns of cervical regionalization across pelecanimorph lineages and tested for vertebral shape differentiation in ardeids, while including anhingas descriptively in morphospace comparisons due to limited sampling. We hypothesized that ardeids are morphologically distinct in one or more cervical regions and that both ardeids and anhingas exhibit unique patterns of cervical regionalization relative to other pelecanimorphs. Our results indicate that (1) ardeids and several lineages within Suliformes, including anhingas, exhibit distinctive patterns of cervical regionalization, and (2) ardeids evolved uniquely shaped vertebrae across cervical regions 1–4 relative to other pelecanimorphs. Anhingas exhibit cervical regionalization patterns similar to those of ardeids, consistent with convergent evolution of neck organization. Additionally, substantial morphological diversity within ardeids may reflect ecological differences in neck use and foraging strategies, highlighting the need for future studies linking cervical form and function. Overall, this study underscores the evolutionary lability of the avian neck and its potential role in facilitating ecological diversification across avian lineages.

## Introduction

Birds exhibit a wide variety of feeding methods that place differing functional demands on the craniocervical system. For example, flamingos use their neck to sweep their head side to side while filter feeding (Jenkin 1957), whereas vultures use forceful neck movements to tear flesh from carcasses (Snively et al. 2014; Böhmer et al. 2020). Correspondingly, avian cervical vertebrae show remarkable morphological variation across species (Boas 1929; Böhmer et al. 2015; Terray et al. 2020; Marek et al. 2021). This diversity spans vertebral number, vertebral shape, and patterns of cervical subregionalization. Cervical counts range from 10 in parrots to 26 in swans (Böhmer et al. 2015), in contrast to the near-constant count of seven in mammals (Galis 1999), and vertebral shapes vary substantially both along the neck and among taxa (Böhmer et al. 2015; Terray et al. 2020; Marek et al. 2021). Recent work has established a five-region framework for the avian neck based on vertebral morphology and developmental patterning (Böhmer et al. 2015; Marek et al. 2021; Marek and Felice 2023), enabling comparative analyses of cervical evolution. Although broad ecological effects on cervical morphology appear limited, vertebrae within individual subregions may reflect adaptations to specialized kinematic demands (Marek et al. 2021). Taxonomically focused studies are therefore needed to resolve fine-scale patterns that may be obscured in large comparative datasets and to generate functional hypotheses about specialized neck morphologies. One avian feeding method thought to place substantial functional demands on the neck is the remarkable ambush predation strategy observed in ardeids and anhingas: a rapid straightening of the neck to propel the head toward prey. Ardeids (family Ardeidae, consisting of herons, egrets, and bitterns) primarily live in wetland habitats, where they opportunistically hunt prey such as fish, invertebrates, amphibians, and small mammals (Recher and Recher 1968; Kushlan 1976). While stalking prey, ardeids often exhibit a slow, deliberate approach. When the prey is within striking distance, the neck rapidly extends and the head is propelled forward (Kushlan 1976). The prey is typically grasped in the bill, but is sometimes impaled depending on the ardeid species and prey size (Mock and Mock 1980). Anhingas, a small group of related but phylogenetically distinct birds, have convergently evolved a similar striking behavior for underwater foraging (Forbes 1882; Boas 1929). Forbes (1882) detailed this behavior, noting that anhingas propel themselves through the water with half-extended wings and paddling feet, capturing small fish with a distinctive, rapid motion of their head and neck similar to a harpoon’s strike. After impaling their prey with their bill, they surface to toss the fish into the air before swallowing. Both ardeids and anhingas belong to the clade Pelecanimorphae, an order that also encompasses storks, frigatebirds, gannets, cormorants, pelicans, ibises, and others (Jetz et al. 2012).

Previous researchers have speculated about form-function relationships in the ardeid and Anhinga cervical column that may enhance feeding performance. A pervasive—yet untested—hypothesis is that a single mid-cervical vertebra, C6 in ardeids and C8 in anhingas, plays a crucial role (Donitz 1873; Forbes 1882; Böker 1937; Král 1965). Building on 19th-century observations of Anhinga feeding behavior, Donitz and Forbes noted that the C7–C8 and C8–C9 articulations allow C8 to flex to produce a Z-shaped bend that could be rapidly straightened during prey capture; Forbes (1882) even illustrated a mechanism emphasizing rotation at C8–C9 (Donitz 1873; Forbes 1882). In 1937, Hans Böker argued that egrets and anhingas (and possibly the sunbittern) independently evolved versions of this configuration for ambush predation and proposed elastic energy storage in the double bend (Böker 1937).

Bohumil Král (1965) was the first to publish a comparison of ardeid to non-ardeid cervical morphology, contrasting the Grey Heron (*Ardea cinerea*) with the White Stork (*Ciconia ciconia*), a tactile forager, and surveying 47 individuals across nine species for skull, vertebral, and muscular anatomy (Král 1965). He asserted that the capacity to form a mid-cervical Z-bend is crucial for rapid strikes, concluding that herons possess adaptations for this behavior whereas storks do not. While influential, these inferences rest on limited, largely one-dimensional measurements and lack a phylogenetic framework. Broader sampling and quantitative, whole-shape analyses are needed to test whether ardeid cervical vertebrae are truly distinctive in form or evolution compared to those of their close relatives.

The present study aims to build on Král’s (1965) investigation by evaluating cervical vertebral shape within a phylogenetic framework using a comparative sample of 24 pelecanimorph species, with particular emphasis on ardeids and anhingas. This sampling captures representatives across the major lineages of the clade, allowing us to assess whether ardeids exhibit osteological differences compared to their close relatives. We test two hypotheses: (1) ardeids exhibit unique regionalization patterns relative to other pelecanimorphs and (2) they possess distinct cervical morphology, especially in the middle cervical region that may be disproportionately involved in strike mechanics. Our approach uses 3D geometric morphometrics and phylogenetic comparative methods to assess evolutionary dynamics of cervical vertebral shape change in each cervical region. The primary strengths of 3D geometric morphometric approaches are their ability to capture shape information in all three spatial dimensions, providing a more comprehensive representation of form, especially for objects with complex, non-planar shapes such as vertebrae (Adams et al. 2004; Zelditch et al. 2012; Cardini 2014). Although both ardeids and anhingas exhibit similar feeding behaviors, the present study primarily focuses on ardeids due to their species-rich phylogeny but gives special attention to the Anhinga where relevant. By elucidating the evolution of cervical vertebral shape in this clade, we aim to provide context for possible functional and ecological significance of neck morphology in Ardeidae, with broader implications for understanding the morphology underlying the diversity of avian feeding behaviors.

## Methods

### Specimen collection

We obtained 23 digital reconstructions of whole birds representing 23 species through MorphoSource.org (Boyer et al. 2016) and one reconstruction of an additional species through the Idaho Virtual Museum (https://virtual.imnh.iri.isu.edu), resulting in a total of 24 species (Supplementary Table S3). All MorphoSource reconstructions were CT or μCT scans except for *A. herodias*, which consisted of high-resolution laser scans of individual vertebrae. Overall, this sampling included 10 ardeids, 1 anhinga, and multiple representatives from the other major lineages (Suliformes, Pelecani, Threskiornithidae) in the Pelecanimorphae clade. Vertebrae from CT scans were segmented in 3D Slicer (Fedorov et al. 2012) and the resulting meshes were cleaned and inspected in Meshmixer (Autodesk 2017). Cervical vertebrae were defined as those without articulated ribs. The atlas (C1) was not included in this study since it is not included as part of the five-region framework of the avian neck.

### Region delineation

Cervical regionalization was determined using an integrative approach that combined qualitative morphological criteria, geometric morphometric analyses, and hierarchical clustering methods. The morphological criteria of Marek et al. (2021) were used initially to qualitatively assess the boundaries between the five cervical subregions for each species and hypothesize the patterns of regionalization for representative species in Ardeidae, Threskiornithidae (ibises and spoonbills), Scopidae (hamerkop), and Balaenicipitidae (shoebill). These criteria include descriptions of neural spine height, centrum length, and depth, and orientations of articular surfaces for each cervical region (Marek et al. 2021).

Next, vertebral shapes were analyzed within species to visualize patterns of shape similarity and discontinuity along the cervical series. We conducted geometric morphometric analyses using an automated pseudolandmarking approach implemented in Auto3dgm (Boyer et al. 2015) within 3D Slicer (Fedorov et al. 2012). Auto3dgm samples each mesh with a dense, approximately uniform set of surface points (“pseudolandmarks”) and then aligns specimens (scale/translation/rotation) to establish point-to-point surface correspondences across shapes. Previous studies have demonstrated that pseudolandmarks recover morphological variation that is consistent with manually landmarked datasets while improving repeatability and reducing observer error (e.g., Boyer et al. 2015, Black and Armbruster 2022). We generated the following numbers of pseudolandmarks for each region: Region 1, 100; Region 2, 300; Region 3, 200; Regions 4–5, 300. The number of pseudolandmarks depended on the approximate minimum number required to properly align vertebrae from each cervical region during the Auto3dgm landmarking process based on initial alignment tests. Region 1 (C2, the axis) was omitted from this analysis, as it is consistently treated as a single-vertebra region in all species included in Marek et al. (2021) and is well established as constituting its own cervical region. Alignment of all meshes within each region was visually inspected. In Region 5, several geometries did not align properly even with high numbers of pseudolandmarks and were not included in the analysis (*A. anhinga, B. rex, N. caledonicus*, and *T. spinicollis*). The pseudolandmarking scheme used for each species, as well as any omitted vertebrae, are shown in Supplementary Materials Table S1. We used generalized procrustes analysis (geomorph 4.0.10; gpagen) in R version 4.1 (R Core Team 2022) to remove the effects of position, size, and orientation of the shapes. We then conducted principal component analyses (PCA) of vertebrae in regions 2–5 for individual species to assess whether vertebrae in different regions clustered separately in morphospace (Supplementary Materials Figure S1).

**Fig. 1:**
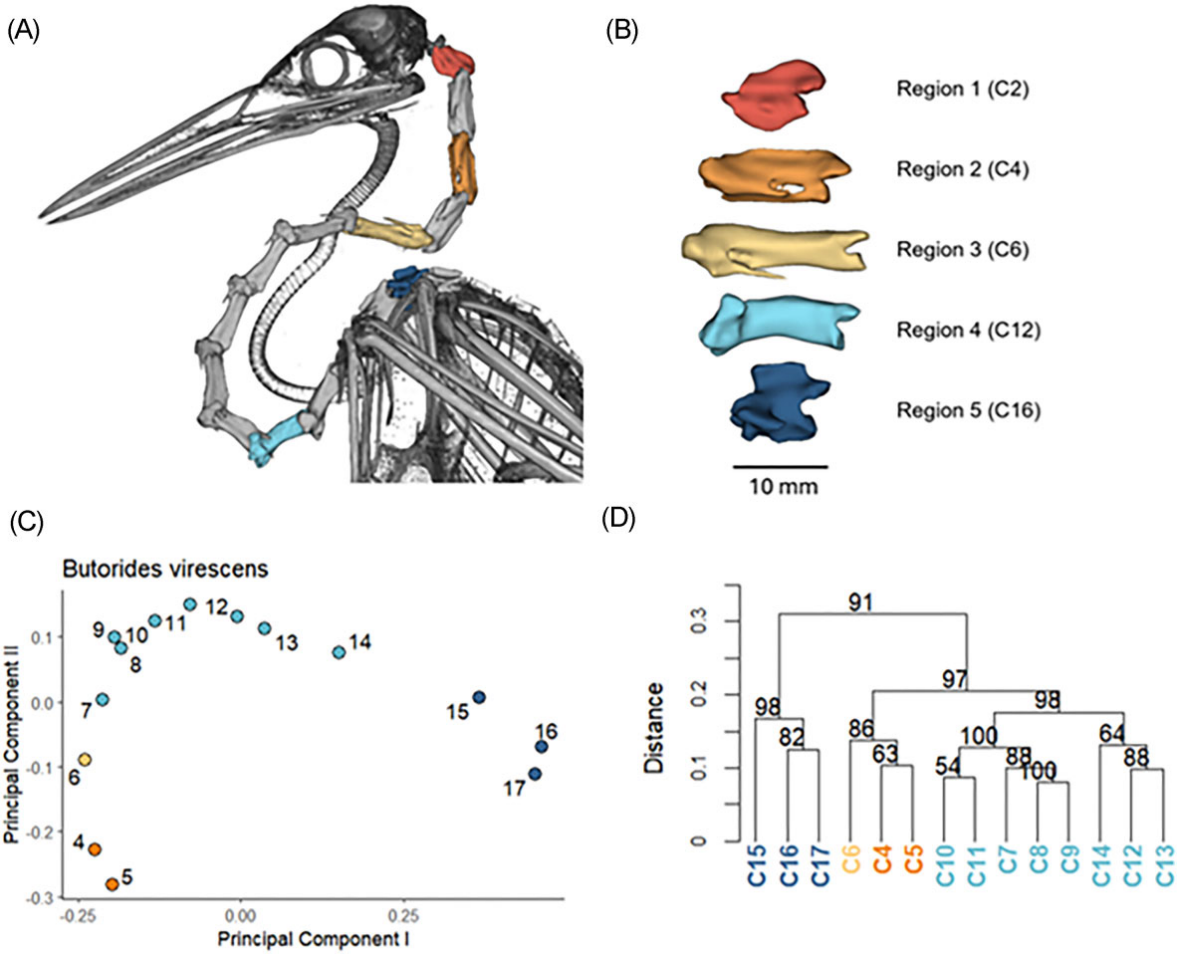
(A and B) Representative vertebrae from the five cervical subregions in a heron neck (*Butorides virescens*). Individual vertebrae are shown in (B), with the cranial end facing left. (C) PCA plot showing PCs 1 and 2 for cervical vertebrae 4–17 of *Butorides*. (D) Hierarchical clustering dendrogram with bootstrap values at the nodes. Specimen: *Butorides virescens*, University of Florida, ID 000S46849.

Following the approach used by Marek et al. (2021), we complemented the PCA with hierarchical clustering dendrograms to visualize patterns of similarity among vertebrae within species and to assist in evaluating hypothesized regional boundaries with bootstrap support. This was done using the hclust() function in base R (Fig. 1C, D). We also evaluated differences between our automated pseudolandmark method and the manual landmark-based delineation of Marek et al. (2021) using the four species common to both studies (Supplementary Table S2). The combined region delineation analyses were used to inform regional assignments and the selection of representative vertebrae for downstream comparative analyses.

### Vertebral sampling strategy

Because cervical regions vary in vertebral number among species, we selected a single representative vertebra from each region containing more than one vertebra to facilitate cross-species geometric morphometric comparisons. We selected representative vertebrae using the following criteria. For Region 2, a representative vertebra was selected at the center of the region. Region 3 vertebrae were selected as the first vertebra in this region in all species, which corresponds to C6 in ardeids and C8 in anhingas. Region 4 vertebrae were selected as those that were two vertebrae cranial to the Regions 4–5 boundary. This caudally-anchored approach for Region 4 was used due to unclear boundaries between Regions 3 and 4 in some species. Selecting vertebrae toward the caudal end of this region minimized the chances of selecting non-homologous vertebrae. Region 5 vertebrae were selected as the vertebra at the center of the Regions 4–5 transition and the first thoracic vertebra. For regions in which a central vertebra was selected, the more caudal vertebra was chosen when the number of vertebrae was even. Examples of representative vertebrae from each region are shown in Fig. 1(A, B), and a complete list of selected vertebrae for each region is available in Supplementary Table S3.

### Phylogeny construction and PhyloPCA

We then performed phylogenetically aligned principal component analyses (phyloPCAs) for each cervical region to identify major axes of shape variation while accounting for shared evolutionary history among species. These phylogenetically aligned PCAs are distinct from the PCAs used for regional delineation and were conducted on the set of representative vertebrae selected for each cervical region. The phylogenetic trees used in all analyses were obtained from BirdTree (http://birdtree.org/) with the Ericson backbone (Jetz et al. 2012) using the “rtrees” package version 1.0.3 (Li 2023). We obtained a posterior sample of 100 time-calibrated trees, then constructed a maximum clade credibility (MCC) tree. The MCC tree represents the tree with the highest clade credibility score. To account for uncertainty in the tree distribution (see Supplementary Fig. S3), we ran our phylogenetic comparative analyses on an alternate tree with the family structure indicated in Kuhl et al. (2021). This phylogeny has the same family structure as that of BirdTree, except that the Pelecani clade (Scopidae, *Balaeniceps*, and Pelecanidae) and Threskiornithidae positions relative to Ardeidae are interchanged. The alternate tree was generated using the move.lineage() function in the RRphylo package version 3.0.0 (Castiglione et al. 2018). The results were robust to the choice of these trees and are summarized in Supplementary Figs. S4–S6 and Supplementary Table S4. We considered PCs explaining at least 10% of the shape variance for interpretation, which constituted PCs 1 and 2 in each region as well as PC 3 in Region 1. We tested whether vertebral shape differs between ardeid and non-ardeid species using multivariate phylogenetically generalized least squares (PGLS) (geomorph 4.0.10; procD.pgls) on Procrustes-aligned landmark coordinates obtained from a generalized procrustes analysis (geomorph 4.0.10; gpagen). This model assumed a Brownian-motion phylogenetic covariance on the time-scaled tree and assessed significance with RRPP permutations (9999). The PGLS analysis tests whether ardeid cervical vertebrae are statistically different from the rest of the Pelecanimorphae clade after accounting for the phylogenetic relationships among species.

## Results

### Region delineation results

PCA and hierarchical clustering analyses showed that vertebrae hypothesized to belong to the same cervical region generally clustered together within species. Regional assignments were largely consistent with those reported by Marek et al. (2021) for the four species common to both studies (Supplementary Table S2). The primary discrepancy involved *Fregata aquila*, in which the boundary between Regions 3 and 4 differed between approaches. Taken together, these approaches revealed separation of vertebrae in morphospace that was largely consistent with our initial hypotheses of regional boundaries based on morphological criteria.

### Cervical regionalization patterns

The total vertebral count varied from 15 in *Scopus* to 18 in *Phalacrocorax*. General patterns consisted of members of Suliformes having more vertebrae in Region 2 and fewer vertebrae in Region 3, except for *Fregata*. The Anhinga, Northern Gannet (*M. bassanus*), cormorants (*Phalacrocorax*), Masked Booby (*S. dactylatra*), and all members of Ardeidae had only one vertebra in Region 3.

**Fig. 2:**
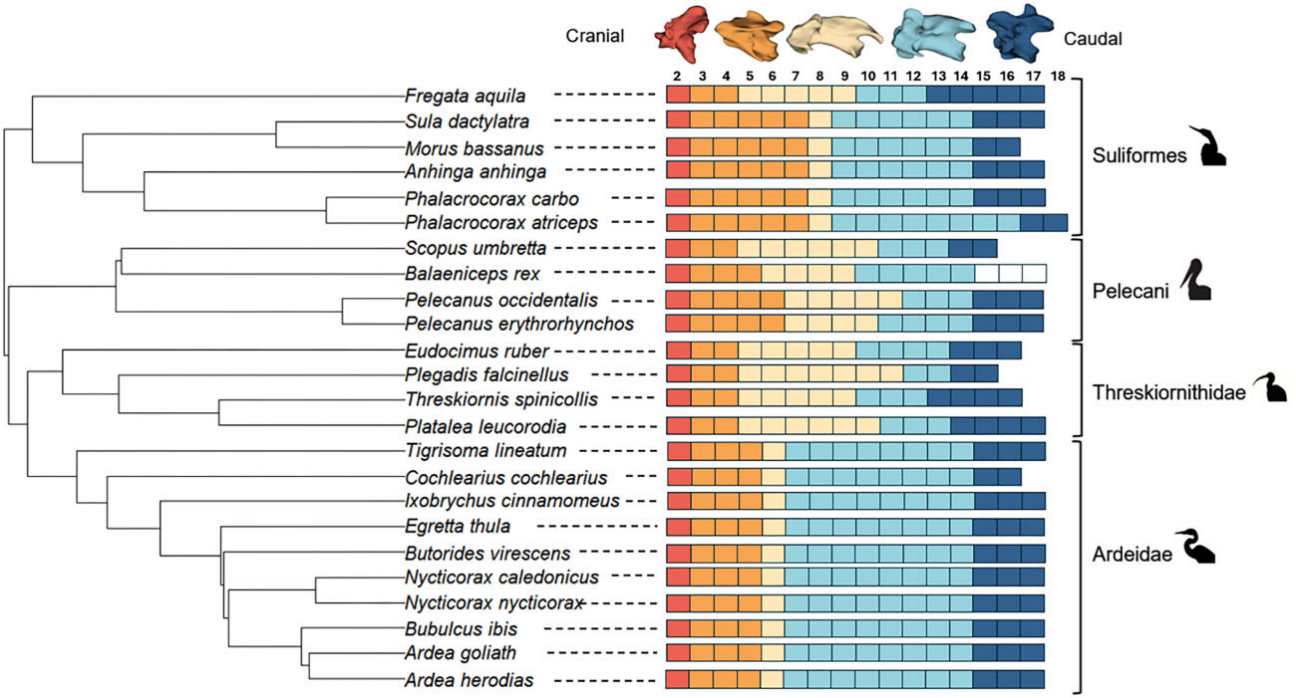
Cervical regionalization in the major branches of pelecanimorph birds. Each square represents a single vertebra and is colored according to its region identity. Region 1: Red, Region 2: Orange, Region 3: Yellow, Region 4: Light blue, and Region 5: Dark blue. Representative vertebrae are shown above the vertebral counts (from *E. ruber*). Data for *B. rex* in the most caudal region of the cervical spine were unavailable and are represented as uncolored boxes.

Across all species, Region 1 consisted of C2. Region 2 varied from 2 to 5 vertebrae, and Region 3 varied from a single vertebra in Ardeidae and Anhingidae to 7 vertebrae in *P. falcinellus*. Region 4 exhibited substantial variation, with 2 vertebrae in frigatebirds (*Fregatidae*) and up to 8 vertebrae in Ardeidae. Region 5 consisted of between 2 and 6 vertebrae, where *Scopus* had the lowest count and *Fregata* had the highest count. These patterns in cervical regionalization are shown in Fig. 2.

### Intraregional variation using 3D shape analysis

**Fig. 3:**
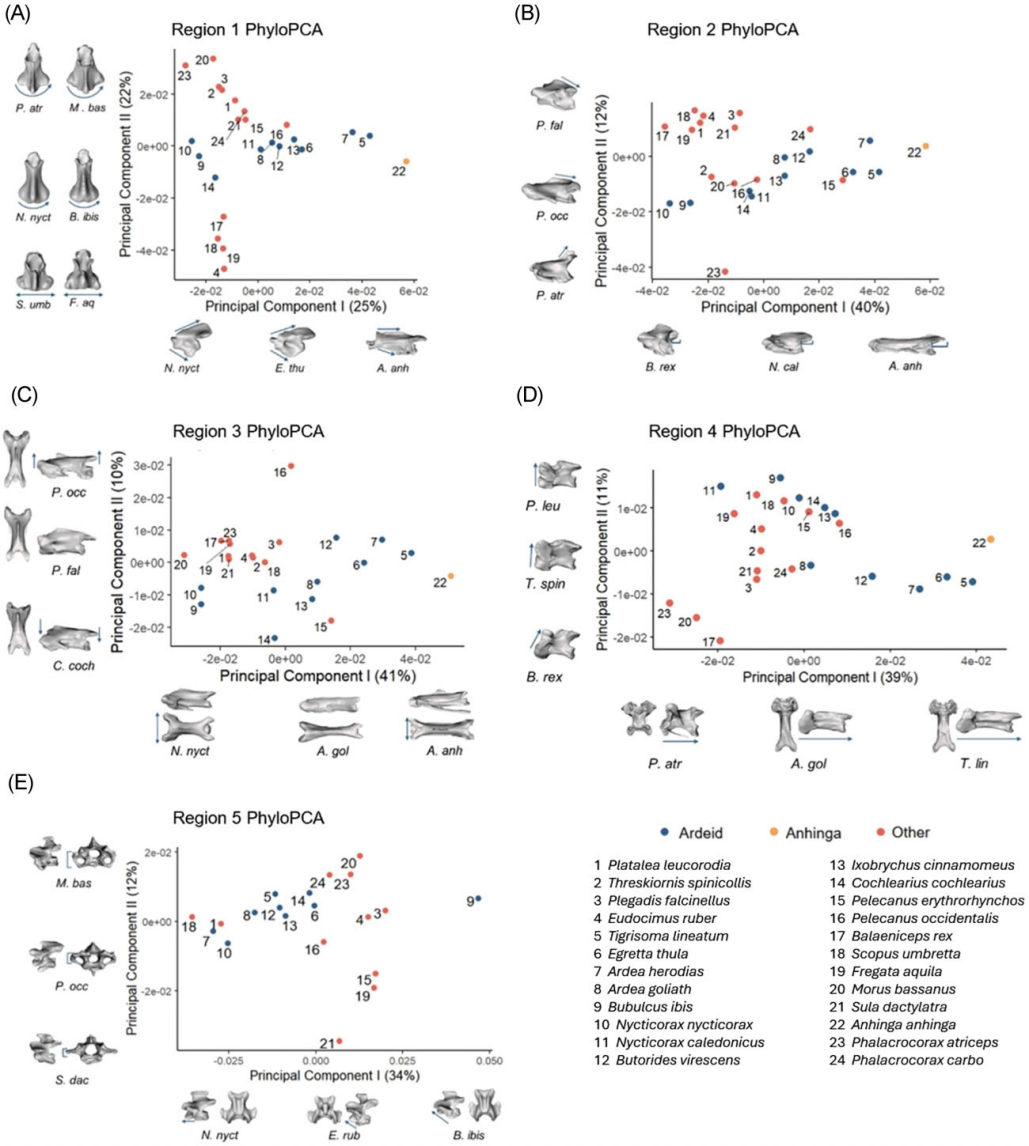
Phylogenetic PC plot of the first two PCs of 3D shape change in Regions 1–5 (A–E, respectively). The vertebral models and small arrows show qualitative observations of shape changes along each PC based on models warped to PC extremes using thin-plate spline. Representative vertebrae from among the included species are provided along each axis for reference. The cranial end of the model vertebrae is facing left for all lateral views, upward in all dorsal views, and facing forward in all transverse views. The legend applies to all plots. The category “Other” includes all species other than ardeids and anhingas.

The patterns of shape variation within each cervical region are visualized in the phylogenetic PCA plots presented in Fig. 3. For each principal component (PC) plot, PC 1 and PC 2 are shown. The only PC explaining at least 10% of the variation that is not shown in Fig. 3 (Region 1 PC3) is presented in Supplementary Fig. S2. Scree plots showing the relative amounts of variation explained by each PC are shown in Supplementary Fig. S1.

### Region 1

In Region 1 (Fig. 3A), PC 1 accounted for 25% of the total variation and captured changes in vertebral length, as well as the height of the neural spine and ventral process, such that more elongated vertebrae appear more streamlined. Ardeid vertebrae spanned a wide range of PC 1 values, demonstrating variability in these features. The Anhinga displayed vertebrae that were even more elongated than those of the ardeids, situated at the far positive end of PC 1. The ardeid species were relatively concentrated in the middle range of PC 2, which accounted for 22% of the observed shape variation and consisted primarily of a change in the shape and orientation of the postzygapophyses. PC 3 (10% of variation) represents a change in the width of the caudal articular surface (Supplementary Fig. S2), with *B. ibis* occupying a unique area of morphospace.

### Region 2

In Region 2 (Fig. 3B), the primary axis of variation accounted for 40% of the total variation, involving changes in vertebral length and the degree of postzygapophyseal protrusion relative to the vertebral body. Ardeids again spanned this axis, with *Nycticorax nycticorax* and *B. ibis* exhibiting some of the shortest vertebrae in the clade. The ardeids *A. herodias, T. lineatum*, and *E. thula* constituted some of the longest vertebrae. *Anhinga anhinga* again had the longest vertebra in this region. Along the axis of PC 2, which accounted for 12% of the variation, the changes included length and dorsoventral orientation of the postzygapophyses.

### Region 3

In Region 3 (Fig. 3C), PC 1 accounted for 41% of shape variation and primarily included length change and mediolateral positioning of the cranial and caudal zygapophyses. Vertebral shapes along the positive end of PC 1 were elongated and laterally compressed compared to those on the opposite end. PC 2 constituted 10% of the total variation and appears to consist mostly of the change in orientation of the postzygapophyses, with morphologies on the negative end of the PC facing more ventrally and those on the positive end more dorsally. Additionally, this PC consisted of a change in the dorsoventral orientation of the prezygapophyses. The ardeids and *Anhinga* again spanned the PC 1 axis, showing that ardeids are diverse in the degree of vertebral elongation in Region 3.

### Region 4

In Region 4 (Fig. 3D), PC 1 (39%) and PC 2 (11%) captured aspects of elongation and orientation of the prezygapophyseal region. The positive end of PC 1 was characterized primarily by elongation, as well as a caudally oriented slant of the prezygapophyseal end when viewed from the lateral perspective. This slanted morphology was the primary shape change represented by PC 2, where the negative end of the PC exhibited a more pronounced slant, and vertebrae on the positive end had a more vertically oriented prezygapophyseal configuration.

### Region 5

In Region 5 (Fig. 3E), length no longer constituted a large portion of the overall variation. Instead, PC 1 (34% of variation) reflected a change in the shape of anterior tubercles ventral to the prezygapophyses. PC 2 (12% of variation) reflected a change in the dorsoventral position of the transverse processes, with more positive values having more ventrally oriented transverse processes. In this region, most ardeids occupied the negative end of PC 1, except for *B. ibis*, which was the only species that occupied the far positive end of this axis.

### Overall shape trends

Overall, the primary ways in which vertebrae differed in shape in Pelecaniformes were the degree of vertebral elongation and orientation or shape of articular surfaces. PC 1 for all regions except for Region 5 consisted primarily of the degree of vertebral elongation. *Ardea herodias, E. thula, T. lineatum*, and *A. anhinga* are consistently on the elongate end of PC 1 for Regions 1–4, with *Anhinga* being at the extreme end of PC 1 in all regions that it was included for analysis. *Nycticorax nycticorax* and *B. ibis* are consistently on the less elongate end of PC 1. *Ixobrychus cinnamomeus* and *A. goliath*, among others, were in the middle range of PC 1 for each region. *Scopus umbretta, B. rex, E. ruber*, and *F. aquila* clustered together in Regions 1–3. *Sula dactylatra* and *B. ibis* occupied their own areas of the morphospace in Region 5.

### PGLS analysis

A summary of the PGLS analysis is shown in Table 1. These results indicate that ardeids are distinct from other members of Pelecanimorphae in cervical Regions 1–4, in which the Procrustes Analysis of Variance (ANOVA) *P*-values are <0.05 (Table 1).

**Table 1:** *P*-values from PGLS analysis and pairwise comparisons

Region	DF	F stat	*R* ^2^	*P*-value
Region 1	2	1.9798	0.15864	*0.020
Region 2	1	2.5335	0.10327	*0.033
Region 3	1	2.7663	0.11640	*0.034
Region 4	1	3.5067	0.13748	*0.014
Region 5	1	2.1437	0.10642	0.089

*Note:* Values with an asterisk are statistically significant (*P* < 0.05).

## Discussion

In this study, we found that (1) several groups in Pelecanimorphae, including ardeids and some members of Sulidae, have unique regionalization patterns compared to their relatives, (2) ardeids have diverse cervical vertebral morphologies that are distinct from other members of Pelecanimorphae in cervical Regions 1–4, but not cervical Region 5. These findings support our hypothesis that unique cervical osteology and regionalization have evolved in ardeids and anhingas. The region-specific patterns of cervical shape differentiation observed in ardeids are consistent with a degree of modularity in the spine. These patterns raise the possibility of modular organization in the avian cervical spine and highlight the need for future analyses designed to test this hypothesis. The diversity in cervical morphology among ardeids may reflect ecological differences in neck use and foraging strategies. Biomechanical studies linking form to function will be necessary for further investigation of this hypothesis.

### Functional hypotheses from intraregional PCA analysis

In Region 1, the ardeids were relatively concentrated in the middle range of PC 2, which consisted primarily of a change in the shape and positioning of the postzygapophyses. This clustering pattern may be due to constraints associated with maintaining joint stability and effective load transmission at the cranio-cervical junction. In Region 4, the primary shape change represented by PC 2 included a caudally oriented slant of the prezygapophyseal end when viewed from the lateral perspective. The increased slant of the prezygapophyseal region may facilitate greater dorsiflexion in the caudal portion of the neck. In Region 5, we found that *B. ibis* was the only species that occupied the far positive end of PC 1, which reflected a change in the shape of cranial tubercles ventral to the prezygapophyses. If a difference in feeding ecology plays a role in the vertebral morphology of this species, it may be due to its more land-based foraging habits of *B. ibis* compared to other members of Ardeidae.

### Potential absence of cervical Region 3

**Fig. 4:**
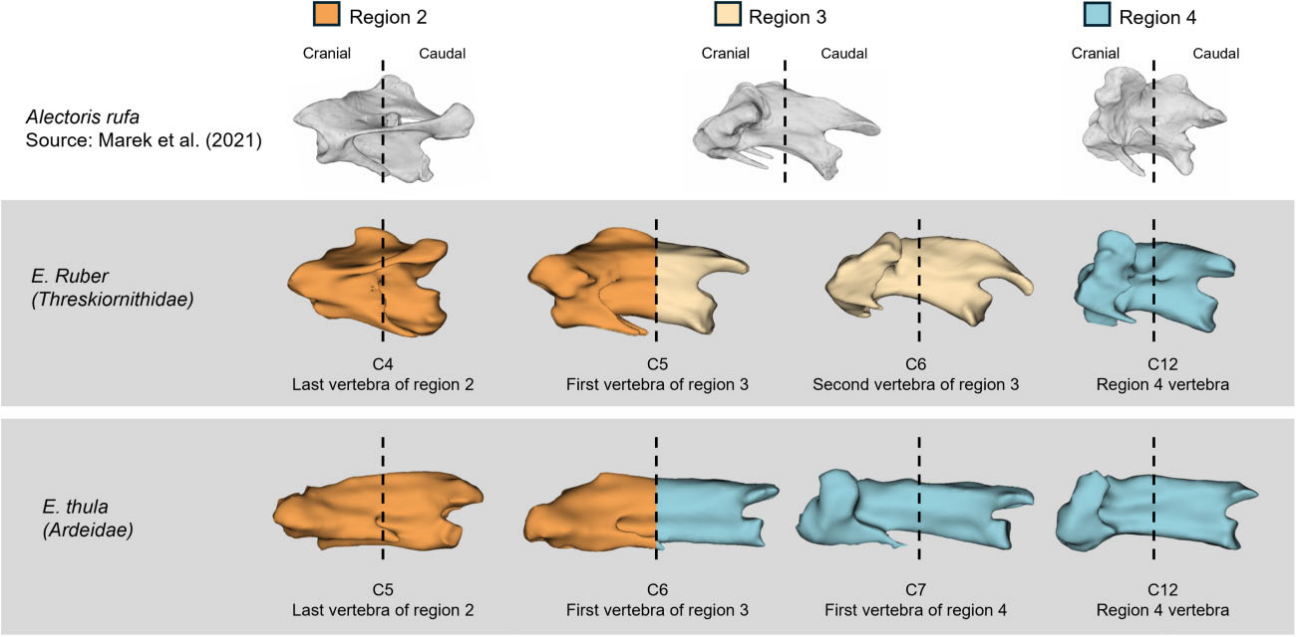
Diagram illustrating how Region 3 may be absent in some members of Pelecanimorphae via qualitative comparison of the cranial and caudal halves of vertebrae in Regions 2–4. Cranial is to the left. Vertebrae with shapes consistent with that of Region 3 described in Marek et al. (2021) (top row) were present in many members of Pelecanimorphae, including the sister group of ardeids (Threskiornithidae, middle row), but absent in others (Ardeidae, bottom row). A similar trend was seen in other species reported here as having a single vertebra in Region 3 (*Anhinga, Phalacrocorax, Sula*, and *Morus*). The vertical dashed lines in the middle of the vertebrae indicate the cranial and caudal halves of each vertebra.

Interestingly, it was unclear whether Region 3 is actually present in ardeids, anhingas, cormorants, and *Morus*, since, in at least some species, the cranial end of these vertebrae resembled the articular surface of Region 2, and the caudal articular surface resembled that of vertebrae in Region 4. Specifically, the caudal portions of these vertebrae lack the pronounced caudoventral curvature of the postzygapophyses that characterizes Region 3 in many other birds and instead exhibit postzygapophyses that extend less caudally beyond the centrum, resembling vertebrae assigned to Region 4. Based on these observations, it is possible that Region 3 is very minimal or even absent in these members of Pelecanimorphae. This hypothesis is illustrated in Fig. 4 and, if supported, would represent a notable departure from the five-region cervical organization currently recognized for birds (Marek et al. 2021; Marek and Felice 2023).

This hypothesis is further supported by vertebral development as each vertebra is formed through sclerotome resegmentation, a process involving the formation of distinct cranial and caudal domains within each sclerotome. These domains subsequently separate and fuse with corresponding domains from adjacent sclerotomes (Gilbert 2003, 448–453). The single vertebra between Regions 2–4 may be more of an intermediate shape rather than its own region. We emphasize that this hypothesis is tentative and would require developmental studies of Hox gene expression for support. Based on the Hox expression boundaries determined by Böhmer et al. (2015), we would predict a change in expression such that there is little to no length of the ardeid neck in which Hox genes A-4 and C-4 are expressed without the presence of Hox gene A-5. Such developmental changes in these groups may have led to the rapid evolutionary rates observed for these same species, since changes in development have been linked to accelerated evolution, especially those involving changes in Hox gene expression (Pick and Heffer 2012). Overall, the regionalization results are intriguing and suggest that, at the regional level, ardeids and some members of Suliformes differ not only from their relatives but from a widespread pattern observed in the rest of Aves (Marek et al. 2021).

### Functional hypotheses from regionalization analysis

The regional patterning found in this study can be used to generate functional hypotheses. For example, the lengthening of Region 4 and shortening of Region 3 effectively results in a longer caudal curvature of the neck. This may result in a longer lever arm over which ventral cervical flexor muscles act, possibly resulting in increased velocity capability during the rapid forward motion used for feeding. Future studies on the relationship between the cranial attachment site and changes in cervical regionalization, in combination with kinematic analyses of neck movements during various activities, are needed to better understand the link between cervical variation and function in Aves.

Another notable pattern that emerged was that, in several members of Suliformes (rather than just ardeids and anhingas) Region 3 is reduced to a single vertebra. We offer potential functional hypotheses for why this regionalization pattern is also present in these species. In *Morus*, a plunge-diver, a shortened, robust mid-cervical segment may reduce buckling risk at water impact. Likewise, in cormorants, a short Region 3 may result in a stiffer mid-neck that minimizes drag-induced oscillations while actively pursuing prey underwater. Frigatebirds often pull prey off the surface or from other birds, so a compact Region 3 might increase torsional rigidity for tugging without buckling.

### Explaining the z-configuration in ardeid and Anhinga necks

Why do ardeids and anhingas have a z-shaped neck appearance, whereas other birds show a smoother S-shaped curvature? Our results, together with previous anatomical studies, suggest that this morphology may be associated with the interaction of cervical regionalization and localized joint mobility, while its external appearance may be influenced by vertebral length and soft-tissue bulk. In ardeids and anhingas, cervical Region 3 is reduced, producing an abrupt transition between Regions 2 and 4. Extra flexion may be facilitated at regional boundaries where vertebral shape changes abruptly, as sudden transitions in articular geometry could locally alter mechanical constraint and permit greater angular excursion. Historical anatomical studies are consistent with this interpretation. Garrod (1876) and Forbes (1882) related increased mobility at the C8 joints to the angular articulation of the eighth cervical vertebra with the seventh and ninth vertebrae, a configuration they described as producing the characteristic mid-neck kink in anhingas. Král (1965) made similar observations about articulations in the mid-necks of ardeids. Birds with higher vertebral counts in Region 3, in contrast, likely distribute bending across more mid-neck joints, resulting in a smoother overall curvature. However, regionalization alone does not fully explain the absence of an externally visible z-shaped configuration in *Phalacrocorax* and *Morus*, which also exhibit a reduced Region 3. A comparable z-shape may be present but obscured by their shorter vertebrae and greater soft-tissue bulk in the neck. Shorter vertebrae make each joint bend less visually pronounced, and thicker, soft tissues smooth the outline of the neck. Direct analyses of joint mobility in these species would be necessary to evaluate this possibility. Ligament architecture likely also influences the magnitude of mid-neck mobility. Boas (1929) and Král (1965) documented that a strong dorsal elastic ligament (ligamentum elasticum interlaminare), present along most of the avian neck, is interrupted in ardeids between C6 and C7, a modification interpreted as permitting greater angular displacement. This localized reduction of dorsal ligamentous constraint may therefore contribute to both enhanced flexibility and the externally distinctive appearance of the ardeid neck.

### Exception to the avian lengthening pattern

In the most caudal cervical region, other aspects of shape variation, such as the shape and orientation of articular processes and transverse processes, were more prominent than length variation. In the context of prior work, this pattern is intriguing. Marek et al. (2021) found that, in birds more broadly, cervical vertebrae tend to lengthen in all regions except 1. Our study shows that some ardeid vertebrae may have become elongated in all regions except 5, demonstrating a deviation from the broader pattern. Future studies of the differences in mechanical demands experienced by different ardeid species may shed light on whether the shape variation seen in these taxa is linked to function.

### Functional hypotheses from 3D shape analysis

Marek et al. (2021) found evidence of differences in vertebral shape between carnivores and insectivores. Carnivores, referred to in the study as those that forcefully tear flesh, tend to have a relatively enlarged, more upright neural spine in the first cervical region. Insectivores, on the other hand, had a shallower neural spine in the fourth cervical region compared to carnivores. Carnivores also displayed more variation in centrum length and height between cervical Regions 3 and 4, while insectivores showed this pattern between cervical Regions 4 and 5. They suggested these differences likely reflect the distinct mechanical demands of carnivory (forceful retraction) vs. insectivory (rapid extension) (Marek et al. 2021). In Ardeidae and the Anhinga, which also feed by rapid extension of the neck, we found that, in Region 1, the neural spine height was variable: The Anhinga had a nearly nonexistent neural spine in C2, but this vertebra in many herons had a more typical shape. Rather, the neural spine is largely absent in Region 2 of ardeids and anhingas compared to other species, resulting in a more streamlined appearance. In the fourth cervical region, it appears that there is not a strong neural spine in Pelecanimorphae in general. Additionally, there was length and height variation observed in ardeids in every cervical region, rather than specific regions. Although there appears to be some overlap in the morphologies of insectivores and rapid strikers, limited knowledge of avian feeding biomechanics limits formal testing of form-function hypotheses, which is a crucial area for future research directions. Integrating biomechanical analyses with morphological data would provide a more comprehensive understanding of the functional significance of observed shape variations.

## Significance

A key question in macroevolutionary and macroecological research is how birds attained exceptional diversity in their ecological roles and physical forms. This study provides insights into the diversity and evolutionary dynamics of avian cervical vertebrae, revealing unique morphologies and neck regionalization in ardeids and anhingas. Most notably, we find that one cervical region (Region 3) may be nearly or entirely absent in certain species, which is a significant deviation from the pattern seen in the rest of Aves and invites questions about the impact of cervical patterning on the neck’s mechanical advantage during feeding.

More broadly, this work suggests a complex evolutionary history of cervical vertebrae within these lineages, with different regions potentially experiencing distinct selective pressures. This conclusion highlights the need for future research to investigate whether specific selective pressures drive regional diversification within various subregions of the neck. Future work incorporating ancestral rate reconstruction could clarify when and where shifts in evolutionary tempo occurred along the avian phylogeny and whether these shifts are consistently associated with changes in cervical regionalization. The morphological framework established here provides a foundation for such analyses.

Collectively, these results provide a foundation for generating testable hypotheses regarding form-function relationships and lay the groundwork for future research exploring the intricate links between cervical anatomy and feeding biomechanics in birds. They also contribute to a growing understanding of avian anatomy and enhance our understanding of the interplay between anatomy, function, and evolution in the axial skeleton.

## Conclusions

This study provides several novel insights into the evolutionary morphology of the ardeid cervical spine. We found that (1) several groups in Pelecanimorphae, including ardeids and several members of the order Suliformes, have unique regionalization patterns compared to their relatives, and (2) ardeids have diverse cervical vertebral morphologies that are distinct from other members of Pelecanimorphae in Regions 1–4, but not Region 5. These findings support our hypothesis that unique cervical osteology and regionalization have occurred in ardeids. Additionally, vertebral morphology was diverse among ardeids themselves, which may reflect ecological differences in neck use and foraging strategies. Another possibility is that ardeids have experienced an evolutionary event resulting in increased morphological diversity, perhaps due to a release of constraints on cervical vertebral shape (Bergmann and Irschick 2012; Goswami et al. 2015; Jones et al. 2018). These data suggest a complex evolutionary history of cervical vertebrae in Ardeidae, with different regions potentially experiencing distinct selective pressures and evolutionary trajectories within the group.

## Supplementary Material

obag004_Supplemental_Files

## Data Availability

The data underlying this article will be available in the Brown Digital Repository Open Data Collection.
