# Supplementary figures and images for: The Peculiar Necks of Herons and Anhingas: A Study of Cervical Morphology in Pelecanimorph Birds
Source: Integr Org Biol. 2026 Feb 12;8(1):obag004. doi: 10.1093/iob/obag004 (PMC13014071; doi:10.1093/iob/obag004)

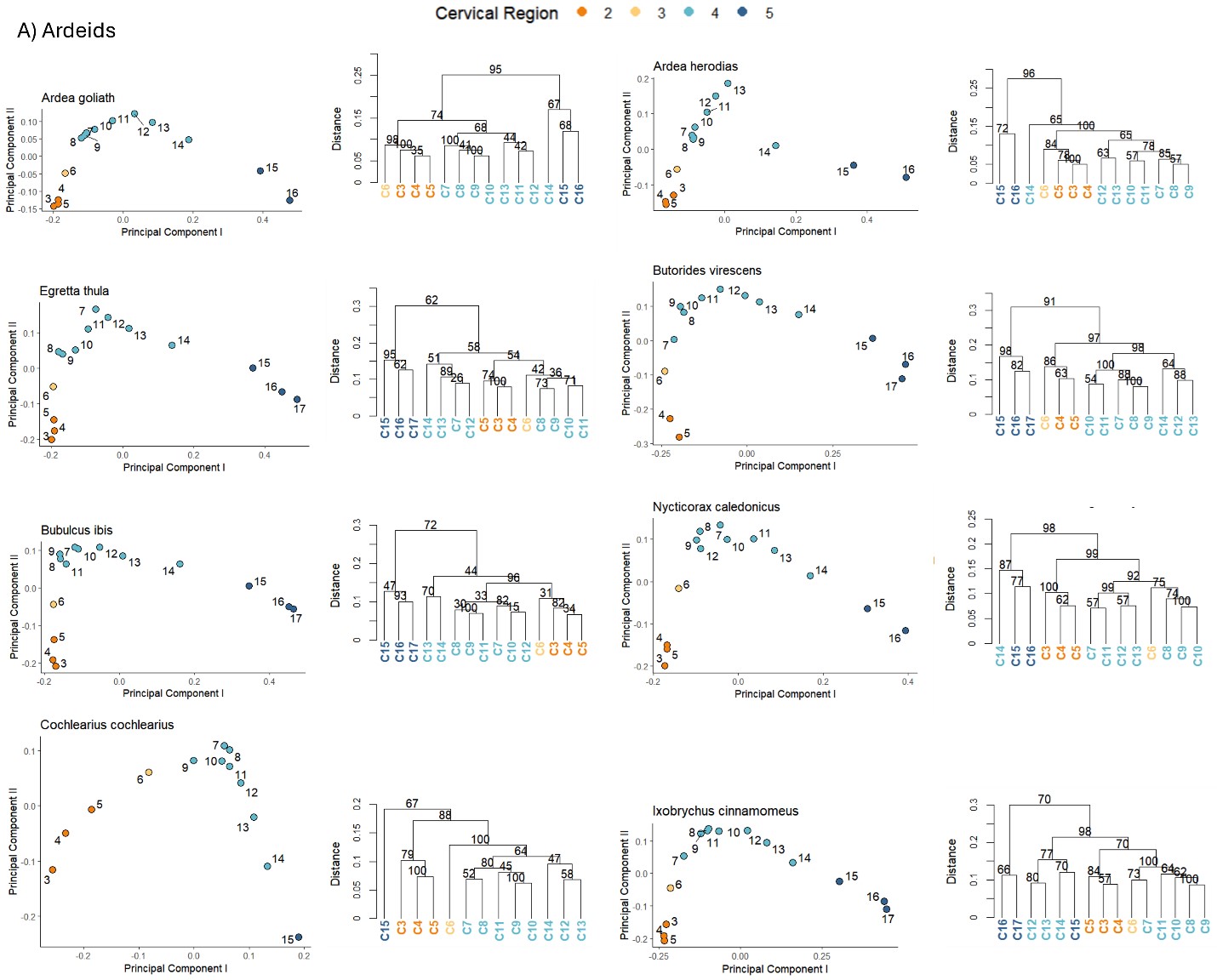

Supplement: obag004_Supplemental_Files [file obag004_supplemental_files.zip › Supplementary_Figure_S1_part1.jpg]

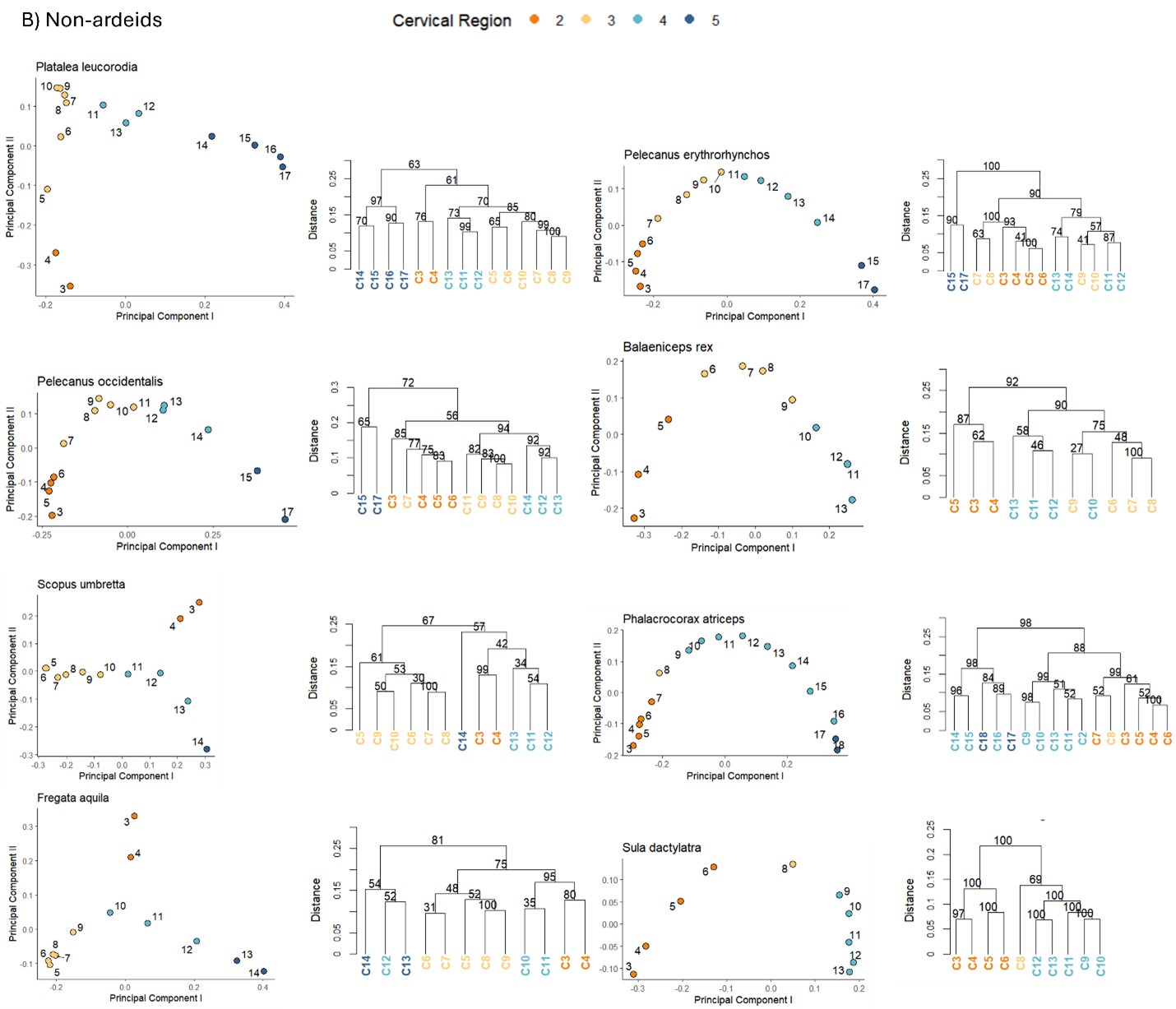

Supplement: obag004_Supplemental_Files [file obag004_supplemental_files.zip › Supplementary_Figure_S1_part2.jpg]

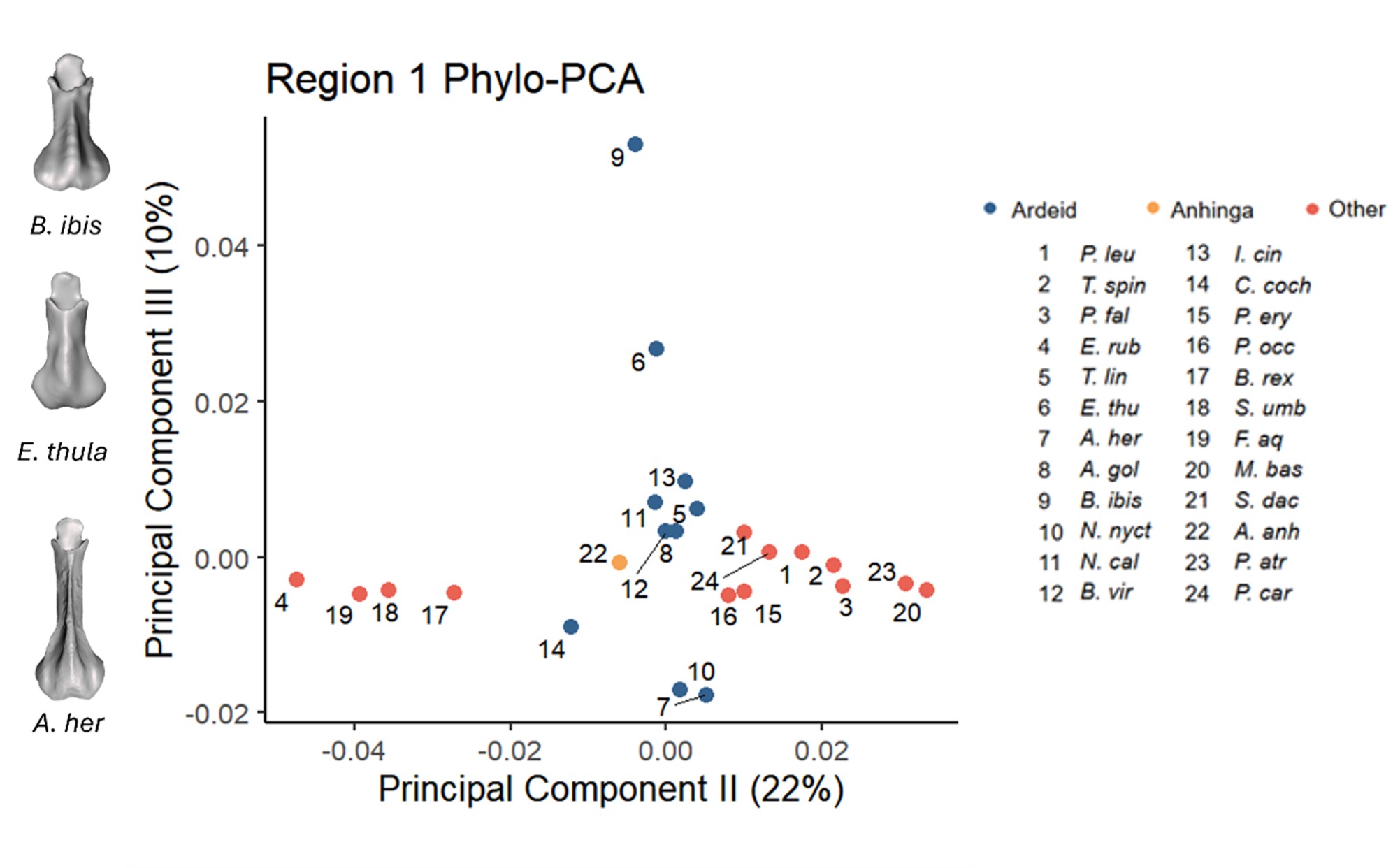

Supplement: obag004_Supplemental_Files [file obag004_supplemental_files.zip › Supplementary_Figure_S2_FINAL.jpg]

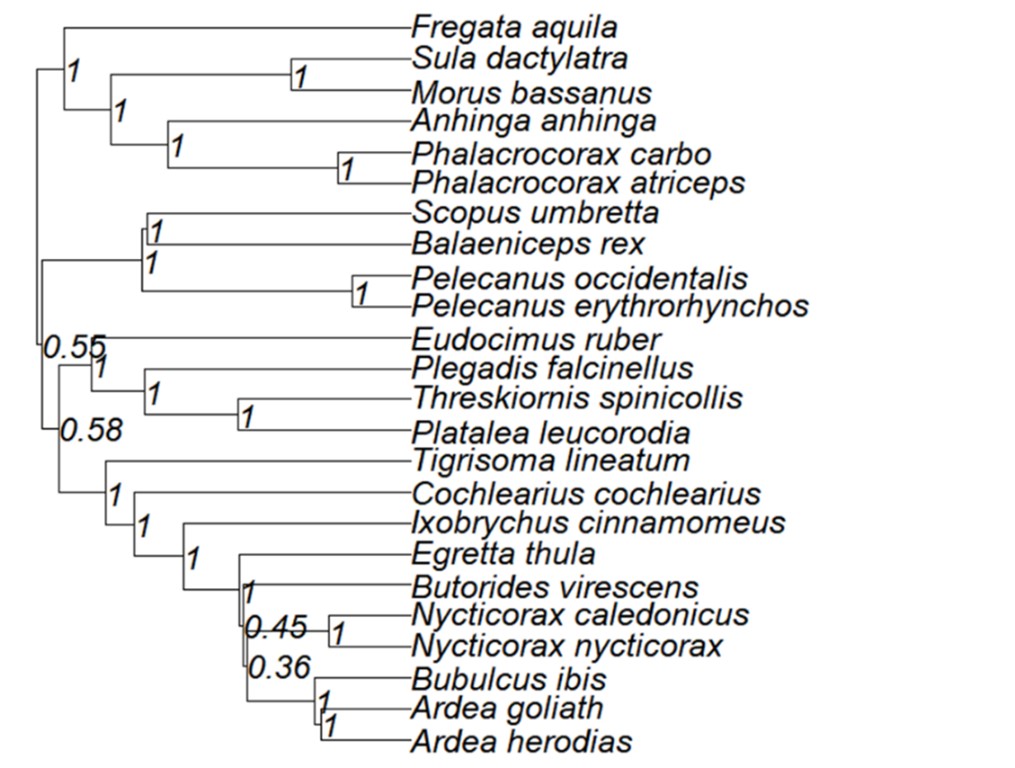

Supplement: obag004_Supplemental_Files [file obag004_supplemental_files.zip › Supplementary_Figure_S3_FINAL.jpg]

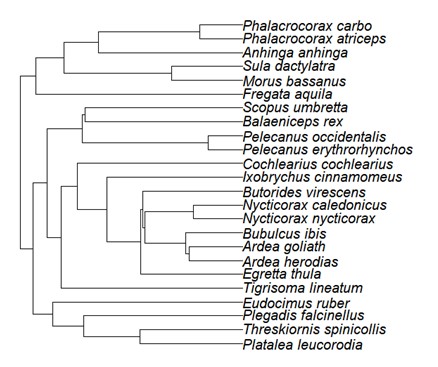

Supplement: obag004_Supplemental_Files [file obag004_supplemental_files.zip › Supplementary_Figure_S4_FINAL.jpg]

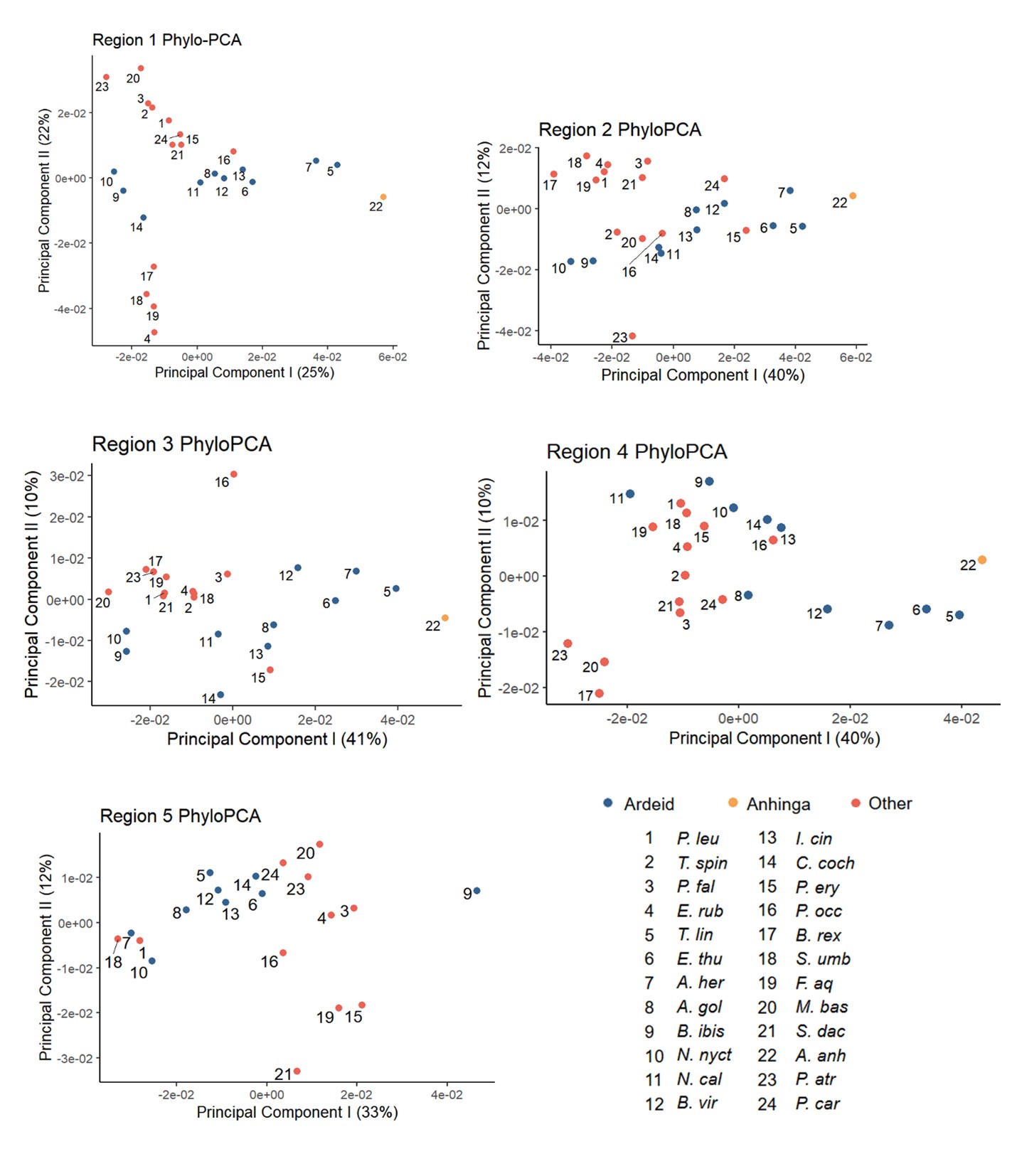

Supplement: obag004_Supplemental_Files [file obag004_supplemental_files.zip › Supplementary_Figure_S5_FINAL.jpg]

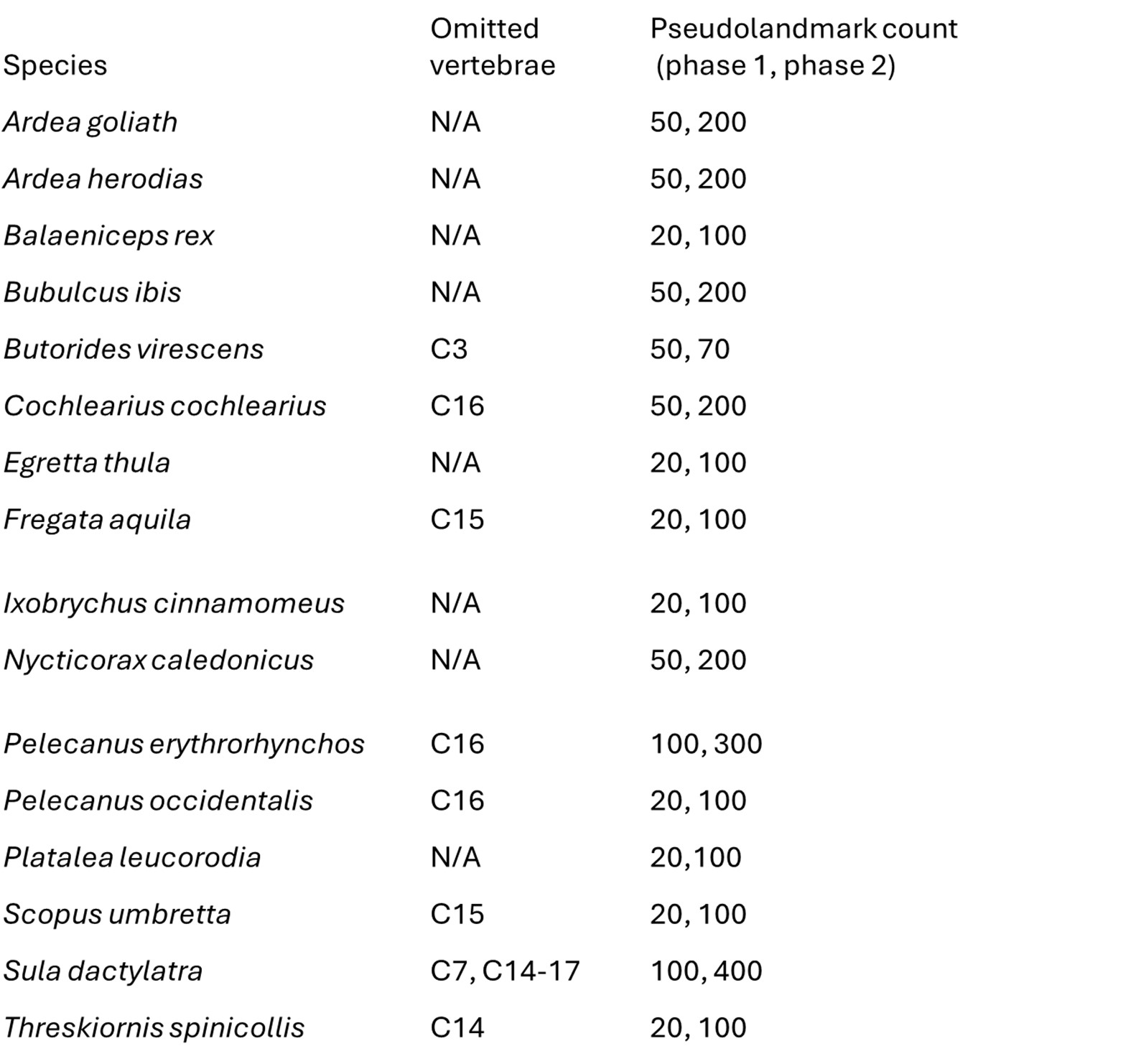

Supplement: obag004_Supplemental_Files [file obag004_supplemental_files.zip › Supplementary_Table_S1_FINAL.jpg]

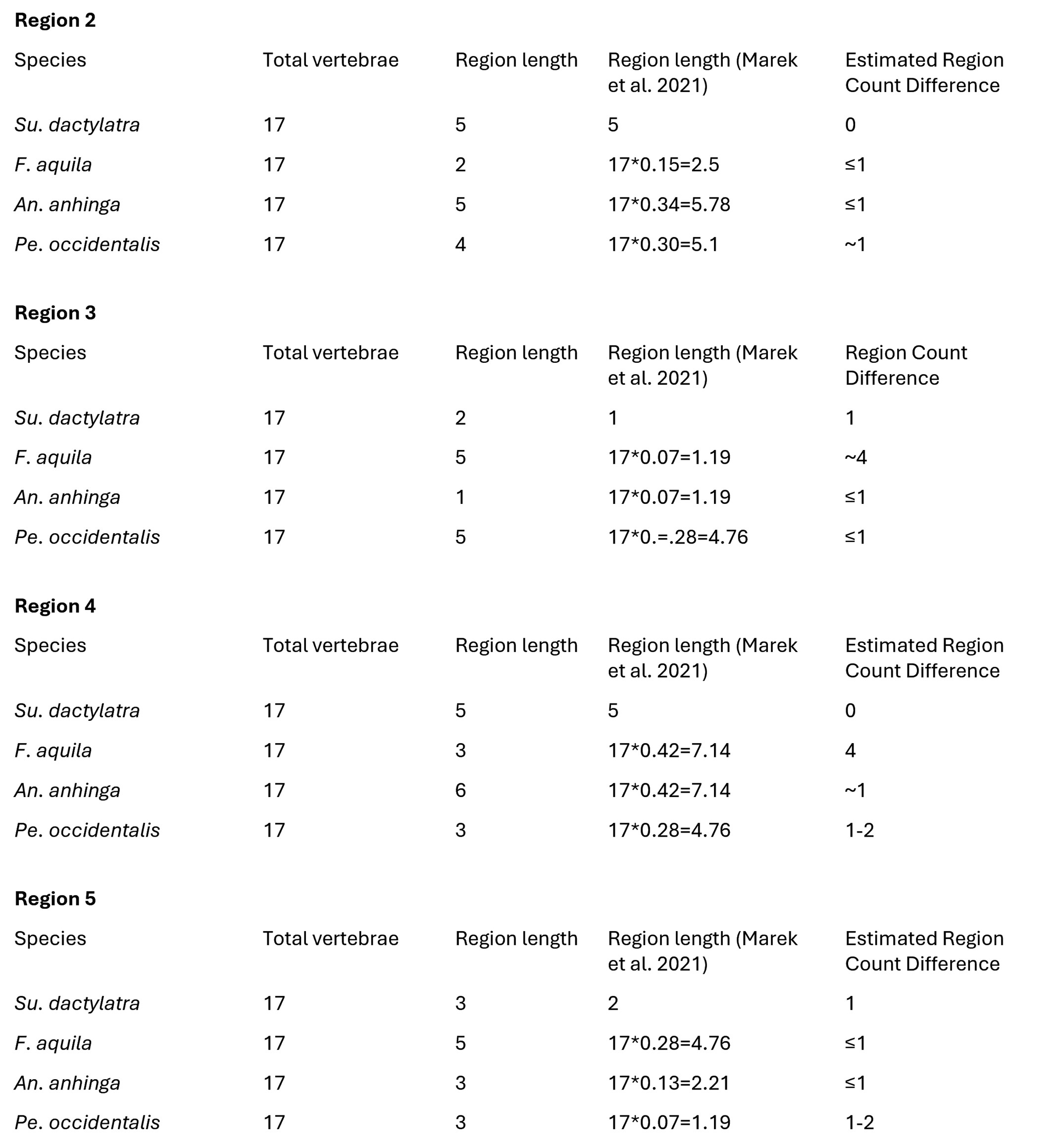

Supplement: obag004_Supplemental_Files [file obag004_supplemental_files.zip › Supplementary_Table_S2_FINAL.jpg]

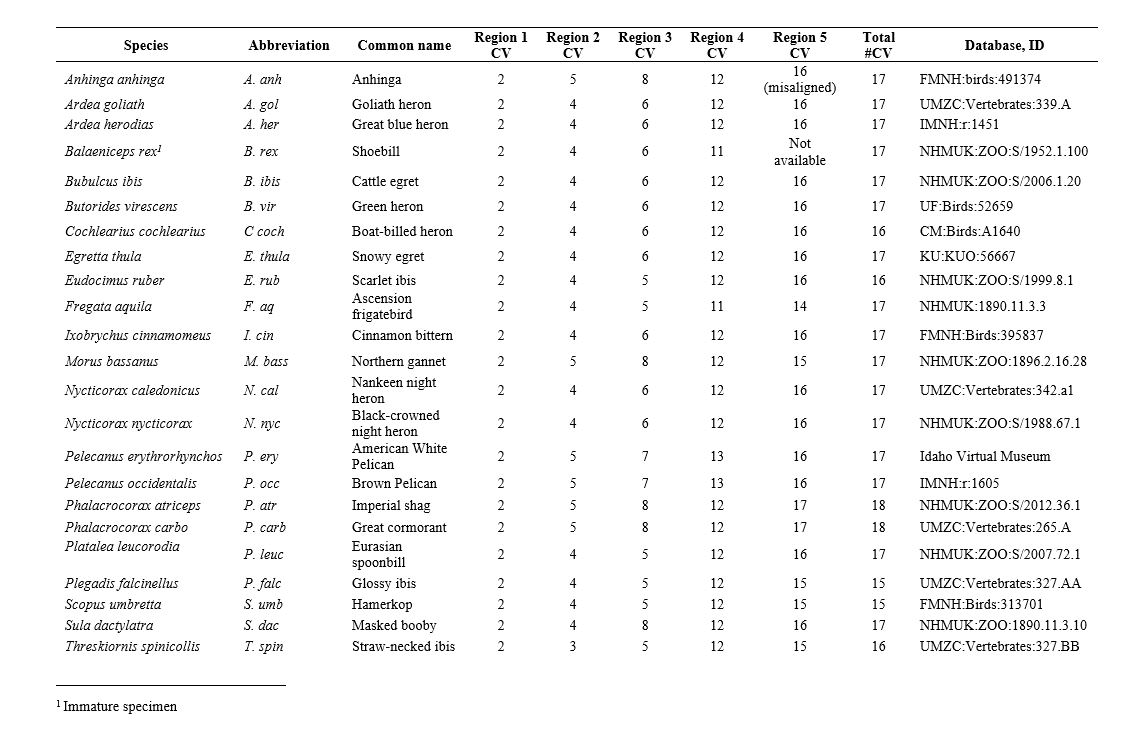

Supplement: obag004_Supplemental_Files [file obag004_supplemental_files.zip › Supplementary_Table_S3_FINAL.JPG]

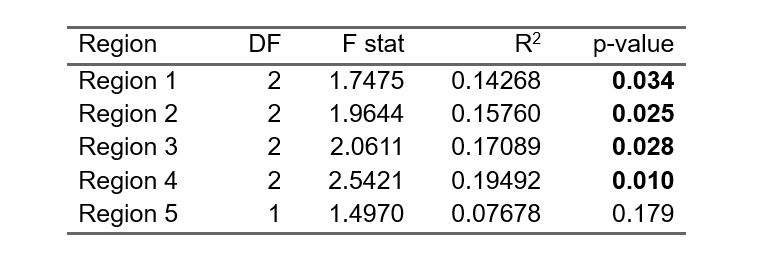

Supplement: obag004_Supplemental_Files [file obag004_supplemental_files.zip › Supplementary_Table_S4_FINAL.JPG]
